# Suppression of TNBC metastasis by doxazosin, a novel dual inhibitor of c-MET/EGFR

**DOI:** 10.1186/s13046-023-02866-z

**Published:** 2023-11-04

**Authors:** Seongjae Kim, Jung Min Park, Soeun Park, Eunsun Jung, Dongmi Ko, Minsu Park, Juyeon Seo, Kee Dal Nam, Yong Koo Kang, Kyoungmin Lee, Lee Farrand, Yoon-Jae Kim, Ji Young Kim, Jae Hong Seo

**Affiliations:** 1 Division of Medical Oncology, Department of Internal Medicine, Korea University College of Medicine, Korea University, Seoul, 02841 Republic of Korea; 2 Brain Korea 21 Program for Biomedical Science, Korea University College of Medicine, Korea University, Seoul, 02841 Republic of Korea; 3 Department of Biomedical Research Center, Korea University Guro Hospital, Korea University, 148 Gurodong-ro, Guro-gu, Seoul, 08308 Republic of Korea; 4 Adelaide Medical School, Faculty of Health and Medical Sciences, The University of Adelaide, Adelaide, South Australia 5000 Australia

**Keywords:** Doxazosin, Triple-negative breast cancer, c-MET, EGFR, Drug accessibility, Metastasis, Cancer stem cells

## Abstract

**Background:**

Triple-negative breast cancer (TNBC) is characterized by aggressive growth and a high propensity for recurrence and metastasis. Simultaneous overexpression of c-MET and EGFR in TNBC is associated with worse clinicopathological features and unfavorable outcomes. Although the development of new c-MET inhibitors and the emergence of 3^rd^-generation EGFR inhibitors represent promising treatment options, the high costs involved limit the accessibility of these drugs. In the present study, we sought to investigate the therapeutic potential of doxazosin (DOXA), a generic drug for benign prostate hyperplasia, in targeting TNBC.

**Methods:**

The effect of DOXA on TNBC cell lines in vitro was evaluated in terms of cell viability, apoptosis, c-MET/EGFR signaling pathway, molecular docking studies and impact on cancer stem cell (CSC)-like properties. An *in vivo* metastatic model with CSCs was used to evaluate the efficacy of DOXA.

**Results:**

DOXA exhibits notable anti-proliferative effects on TNBC cells by inducing apoptosis via caspase activation. Molecular docking studies revealed the direct interaction of DOXA with the tyrosine kinase domains of c-MET and EGFR. Consequently, DOXA disrupts important survival pathways including AKT, MEK/ERK, and JAK/STAT3, while suppressing CSC-like characteristics including CD44^high^/CD24^low^ subpopulations, aldehyde dehydrogenase 1 (ALDH1) activity and formation of mammospheres. DOXA administration was found to suppress tumor growth, intra- and peri-tumoral angiogenesis and distant metastasis in an orthotopic allograft model with CSC-enriched populations. Furthermore, no toxic effects of DOXA were observed in hepatic or renal function.

**Conclusions:**

Our findings highlight the potential of DOXA as a therapeutic option for metastatic TNBC, warranting further investigation.

**Supplementary Information:**

The online version contains supplementary material available at 10.1186/s13046-023-02866-z.

## Background

Triple-negative breast cancer (TNBC) is an extremely aggressive and challenging subtype of breast cancer, representing approximately 15-20% of all diagnosed cases. Standard treatment for TNBC patients still involves cytotoxic chemotherapy due to the absence of well-established molecular targets [1]. TNBC patients have a higher recurrence rate at an average of 1.2 years after initial treatment [2]. Approximately 46% of TNBC patients develop metastases, with a median overall survival of only 13.3 months [3, 4]. TNBC is a molecularly heterogeneous disease characterized by the interplay of complex signaling networks, including the PI3K/AKT, JAK/STAT3, Ras/MAPK and EGFR/c-MET pathways, posing a major challenge for TNBC therapy [1].

Oncogenic c-MET is activated upon binding a pleiotropic factor-like cytokine, hepatocyte growth factor (HGF), which increases cell proliferation, cell proliferation, motility, invasion, and dissemination [5]. Knock-in mice with constitutively activated-c-MET develop basal type-mammary adenocarcinomas, marked by the absence of progesterone receptor (PR) and HER2, and expression of the basal marker cytokeratin 5 [6]. Several c-MET inhibitors including foretinib and cabozantinib are undergoing clinical trials for TNBC patients, but none have been approved by the FDA to date [7].

EGFR is a prognostic determinant for TNBC and is overexpressed in more than 40% of patients [8]. EGFR facilitates tumor cell survival and metastasis by activating signal transduction cascades, including MAPK, AKT and STAT3 [9]. However, the clinical benefit of EGFR-targeted monoclonal antibodies or tyrosine kinase inhibitors (TKIs) has been limited in TNBC [10]. Clinical and preclinical studies highlight a significant interplay between c-MET and EGFR expression in TNBC. Resistance to anti-EGFR TKI monotherapy has been linked to c-MET overexpression, while c-MET TKI monotherapy triggers upregulation and phosphorylation of EGFR, suggesting compensatory receptor tyrosine kinase (RTK) signaling in TNBC and NSCLC [11, 12]. In this respect, future directions should focus on dual blockade or combination strategies targeting both EGFR and c-MET to overcome TKI resistance [12, 13].

90% of deaths from cancer are associated with metastasis [14]. Cancer stem cells (CSCs) are a major driver of tumor recurrence and propagation that can resist conventional chemotherapy and radiotherapy [15]. The removal of these unique cell subsets with heterogeneous features from the primary tumor is required to improve cancer prognosis [1, 16]. Recent evidence suggests that the relationship between CD44 and EGFR observed in CSC clustering promotes TNBC metastasis [17]. Clinical studies have shown that the expression levels of EGFR and CD44 are relatively higher in TNBC compared to other tumors, and patients with both EGFR and CD44 positivity have the worst outcomes for overall survival and disease-free survival [18]. Disruption of the EGFR/CD44 axis therefore represents a promising therapeutic strategy to prevent metastasis in TNBC.

Although novel c-MET (capmatinib, tepotinib) and EGFR inhibitors (osimertinib, lazertinib) have received limited approval for the treatment of various cancers, their high cost limits patient accessibility, particularly in the developing world. Existing compounds that can inhibit these clinically-validated targets may help to address this unmet need. Doxazosin (DOXA) is a quinazoline-based alpha 1-adrenergic receptor (A1AR) antagonist and is a widely-available drug used to treat benign prostate hyperplasia (BPH) and hypertension [19]. For the first time, we report the antitumor efficacy of DOXA in TNBC and explore its potential as a drug repurposing candidate.

## Methods

### Reagents, materials and antibodies

Doxazosin mesylate, crizotinib, capmatinib, tepotinib, osimertinib and lazertinib were purchased from Selleckchem (Radnor, PA). Amivantamab was obtained from MedchemExpress (Monmouth Junction, NJ). Triton X-100, propidium iodide, PBS tablet, dimethyl sulfoxide (DMSO) and cOmplete™ protease inhibitor cocktail were obtained from Sigma-Aldrich (St. Louis, MO). The immunoblotting and immunostaining antibodies utilized were obtained as follows: c-MET, phospho-c-MET (Y1234/1235), EGFR, phospho-EGFR (Y1068), MEK, phospho-MEK (S217/221), AKT, phospho-AKT (S473), cleaved caspase-3 (Asp175), cleaved caspase-7 (Asp198), cleaved caspase-8 (Asp391), PARP, JAK2, CD44, OCT4 and SOX2 (Cell Signaling Technology, Beverly, MA); phospho-JAK2 (Y1007/1008), STAT3, phospho-STAT3 (Y705) and P-glycoprotein (Abcam, Cambridge, MA); survivin, cyclin D1 and ALDH1A1 (Santa Cruz Biotechnology, CA); GAPDH (Invitrogen, Carlsbad, CA). The secondary antibodies used were HRP-conjugated anti-rabbit and mouse IgG (Bio-Rad Laboratories Inc, CA) and Alexa Fluor-488 or -594 goat anti-mouse and rabbit IgG (Invitrogen).

### Breast cancer cell culture

The human TNBC cell lines MDA-MB-231 (PerkinElmer Inc., CT) and BT549 (JCRB Cell Bank, Japan), and the murine mammary carcinoma 4T1-Luc (JCRB Cell Bank) were cultured in MEM or RPMI 1640 (Gibco, Gaithersburg, MD) supplemented with 10% FBS and streptomycin-penicillin (100 U/ml) at 37℃ with 5% CO_2_. All cell lines were passaged for less than 6 months after resuscitation and were used from passages 3 to 20. All cell lines were authenticated by short tandem repeat profiling by Macrogen Inc (Seoul, South Korea).

### Cell viability assay

To examine the anti-proliferative effect of DOXA, the CellTiter 96® Aqueous One Solution Cell Proliferation Assay utilizing 3-(4,5-dimethylthiazol-2-yl)-5-(3-carboxymethoxyphenyl)-2-(4-sulfophenyl)-2H-tetrazolium (MTS) was employed, following the manufacturer's instructions (Promega, WI). The quantification of the formazan product was performed by measuring the absorbance at 490 nm using a Spectramax Plus 384 microplate analyzer (Molecular Devices, CA).

### Cell cycle analysis and Annexin V/PI assay

To explore the impact of DOXA on apoptosis, the Sub-G1 assay, as well as early and late cell death, were evaluated using Annexin V/PI staining. Cells were collected, fixed in 95% ethanol containing 0.5% Tween-20 for 24 hours, and subsequently treated with 50 μg/ml propidium iodide and 50 μg/ml RNase at room temperature for 30 min. For the Annexin V/PI assay, cells were stained with the FITC Annexin V Apoptosis Detection Kit I (BD Biosciences, NJ) following the manufacturer's instructions. The stained cells were then subjected to flow cytometry analysis using a BD LSRFortessa^TM^ X-20 Cell Analyzer (BD Biosciences).

### Analysis of CSC-like properties

ALDH1 activity was assessed using the Aldefluor assay kit (Stemcell Technology, Vancouver, BC). Cells were incubated at 37℃ for 45 min in Aldefluor assay buffer containing the ALDH1 protein substrate BODIPY-aminoacetaldehyde (BAAA, 1 µM per 0.5×10^6^ cells). To establish the baseline for Aldefluor-positive populations in flow cytometry, a specific inhibitor of ALDH1, diethylamino-benzaldehyde (DEAB), was used at a concentration of 50 mM. For CD44^high^/CD24^low^ and CD49f^high^/CD24^high^ staining, cells were incubated at 4℃ for 30 min with FITC- and PE-conjugated anti-mouse IgG or FITC-conjugated anti-CD24 and PE-conjugated anti-CD44 or CD49f antibodies (BD Biosciences) and analyzed by flow cytometry.

### Mammosphere formation assay

To assess the effect of DOXA on mammospheres formation, characterized by self-renewal ability, was evaluated using anchorage-independent serum-free culture conditions. 4T1 (0.2×10^4^/ml) and BT549 (0.7×10^4^/ml) cells were plated in ultralow attachment dishes (Corning, NY) and cultured, as previously described [20]. The number and volume of the mammospheres were analyzed using an Olympus CKX53 microscope. The 3D spheroid volumes were calculated using the formula Volume=4/3*3.14(π)*r^3^ (r: radius).

### Molecular modeling and docking analysis

Molecular docking studies were performed using the GalaxySagittarius software (https://galaxy.seoklab.org/). After completion of the docking simulation, visualization of the 2D and 3D protein-ligand complexes and predicted binding affinity and energy were analyzed using UCSF chimera 1.16 (https://www.cgl.ucsf.edu/chimera/), BIOVIA Discovery Studio 2021 (https://discover.3ds.com/discovery-studio-visualizer-download/) and DockThor web server (https://dockthor.lncc.br/v2/).

### Immunoblot analysis

Cells were lysed in a lysis buffer [30 mM NaCl, 0.5% Triton X-100, 50 mM Tris-HCl (pH 7.4)] supplemented with phosphatase and protease inhibitor cocktail, and incubated on ice for 45 min. Supernatant was collected after centrifugation (14,000 g, 4℃, 20 min) and protein concentrations were determined using a Bradford protein assay kit (Bio-Rad). Equal amounts of protein (25 µg) were separated by SDS-PAGE and electro-transferred onto a polyvinylidene fluoride (PVDF) membrane (Millipore, St. Louis, MO). The membranes were then incubated overnight at 4℃ with primary antibodies diluted in 5% BSA [c-MET (1:2000), phospho-c-MET (1:1000), EGFR (1:2000), phospho-EGFR (1:1000), AKT (1:2000), phospho-AKT (1:2000), MEK (1:2000), phospho-MEK (1:2000), ERK (1:2000), phospho-ERK (1:2000), PARP (1:2000), cleaved caspase-3 (1:2000), cleaved caspase-7 (1:2000), JAK2 (1:2000), phospho-JAK2 (1:1000), STAT3 (1:2000), phospho-STAT3 (1:1000), cyclin D1 (1:2000), survivin (1:2000), ALDH1A1 (1:2000), CD44 (1:2000), Oct-4 (1:2000), Sox-2 (1:2000), P-glycoprotein (1:1000) and GAPDH (1:15,000)], followed by incubation with HRP-conjugated anti-mouse and rabbit IgGs (1:1000-1:20,000). Signal intensity was detected using a chemiluminescence kit (Thermo Fisher Scientific Inc., Rockford, IL) and visualized on X-ray film (Agfa Healthcare, Mortsel, Belgium). Quantification of the signal intensity was performed using AlphaEaseFC software (Alpha Innotech, San Leandro, CA).

### Immunoprecipitation assay

To examine the impact of DOXA on the interaction between EGFR and CD44, the Dynabeads™ Protein G Immunoprecipitation Kit (Thermo Fisher Scientific Inc., Rockford, IL) was utilized following the manufacturer's instructions. Cells were lysed in a lysis buffer (Pierce® IP) containing a cocktail of phosphatase and protease inhibitors. Supernatant was collected after centrifugation (14,000 g, 4℃, 10 min) and equal amounts (1000 µg) were incubated with 4 µg of anti-EGFR antibody conjugated to Dynabeads Protein G at 4℃ overnight. The protein complexes were recovered by boiling the beads in a mixture of SDS-PAGE sample buffer and elution buffer (1:1). Immunoblotting was conducted for EGFR (1:2000) and CD44 (1:2000) using equal amounts of protein (100 µg).

### Immunocytochemistry

Immunocytochemistry was conducted to assess the expression and co-localization of EGFR and CD44 in MDA-MB-231 cells. Cells in Falcon^®^ chambered cell culture slides (BD Biosciences) were fixed with 4% paraformaldehyde, washed with PBS, and incubated with 0.2% Triton X-100 for 13 min. Primary antibodies were applied to the cells in antibody-diluent (Dako, Denmark) were incubated overnight at 4°C. For secondary antibody reactions, Alexa Fluor®-488 or -594 conjugated secondary antibodies (Thermo Fisher Scientific) were used for staining and then mounted with ProLong^®^ Gold Antifade Reagent with DAPI (Thermo Fisher Scientific). Imaging of the cells was performed using a Carl Zeiss confocal microscope (Weimar, Germany), and the intensity of the EGFR and CD44 signal was analyzed using the intensity profile tool.

### Real-time quantitative polymerase chain reaction (RT-qPCR) analysis

Total RNA was extracted using an RNase mini kit (Qiagen, Valencia, CA, USA). The cDNA was synthesized from total RNA using oligo-dT random primers and SuperScript^TM^ III Reverse Transcriptase (Invitrogen) according to each manufacturer’s protocol. The primer sets for cyclin D1, survivin, vimentin, MMP-2, MMP-9, VEGF, Smad-3, Smad-4, P-glycoprotein and GAPDH used for RT-qPCR are listed in Supplementary Table S1. The reaction volume of the RT-qPCR was 20 µL, containing 10 µL of Power SYBR™ Green PCR Master Mix (Thermo Fisher Scientific, Waltham, MA, USA), 1 µL of forward primer (0.2 µM), 1 µL of reverse primer (0.2 µM), 2 µL of cDNA solution, and 6 µL dH_2_O. PCR was carried out using QuantStudio 6 Flex (Applied Biosystems, CA) with QuantStudio^TM^ Real-Time PCR Software under the following conditions: after an initial denaturation at 95ºC for 10 min, cDNA amplification was performed at 95ºC for 15 sec and 60ºC for 1 min for 40 cycles. The relative mRNA levels in the cDNA samples were calculated based on the comparative Ct method (ΔΔCt) with the normalization factor of GAPDH.

### Allograft in vivo experiments and in vivo bioluminescence imaging (BLI)

All animal procedures were conducted in compliance with the guidelines for animal care and approved by the Korea University Institutional Animal Care and Use Committee (IACUC, KOREA-2021-0070). Female BALB/c mice, aged five weeks, were obtained from the NARA Biotech Animal Center (Seoul, Korea), housed in a pathogen-free environment, and acclimated for 1 week prior to the study with free access to food and water. Following acclimation, 1×10^5^ cells from 4T1 mammospheres were injected into the fourth mammary fat pads of 6-week-old BALB/c mice (*n*=5/each experimental group). When average tumor volumes reached 50 mm^3^, the animals were randomized into 2 groups (*n*= 5/each group), and vehicle (DMSO/corn oil, 1:9) or DOXA (5 mg/kg/day) was administered intraperitoneally every other day for 28 days. Tumor volumes were measured twice a week after the initial treatment and calculated using the following formula; V=(Length×Width^2^)/2. After a 48-hour interval following the last administration of DOXA, the mice were then anesthetized and subjected to NightOWL II LB 983 In Vivo BLI System (Berthold Technologies, TN). For in vivo imaging, a chemiluminescent luciferase substrate, D-luciferin sodium salt (Abcam) was administered intraperitoneally at a dose of 150 mg/kg body weight in 100 µL PBS prior to imaging. The captured images were quantified using the IndiGo™, and the quantification was performed in photons per second (photons/sec). For lung metastasis area analysis, lungs were collected, fixed in 4% paraformaldehyde, and embedded in paraffin blocks. 5-μm thick tissue sections were mounted on positively charged glass slides and tissues were stained with hematoxylin and eosin (H&E). The images were taken using a slide scanner, Zeiss Axio Scan.Z1, and areas of distant metastatic lesions/lungs were analyzed with ZEN software. For the syngeneic mouse model of experimental metastasis, 1×10^5^ cells from 4T1 mammospheres were injected into the tail vein of BALB/c mice, followed by a single dose of intravenous control solvent or DOXA (5 mg/kg).

### Immunohistochemistry and in-situ localization of apoptosis (TUNEL)

After removal, tumors were fixed in 4% paraformaldehyde (PFA) and then embedded in paraffin. Tissue sections with a thickness 5 µm were mounted on positive charged microscope slides. Subsequently, the slides were deparaffinized with xylene and dehydrated through a series of graded alcohol solution. Antigen retrieval was performed by boiling the tissue sections in citrate buffer (pH 6.0). Tissue sections with primary antibodies (Ki-67; 1:200, cleaved caspase-3; 1:100, CD31; 1:100, c-MET; 1:100, EGFR; 1:150, CD44; 1:300, ALDH1A1; 1:75, phospho-STAT3; 1:100, Vimentin; 1:200, phospho-c-MET; 1:100, phospho-EGFR; 1:100) in antibody-diluent were incubated overnight at 4°C. For secondary antibody reactions, Alexa Fluor®-488 or -594 conjugated secondary antibodies were applied to tissue sections for staining, incubated at room temperature for 2 h, and mounted with DAPI. *In situ* TUNEL assays were carried out on tissue sections using an *In situ* Cell Death Detection Kit (Roche Applied Sciences, GER) in accordance with the manufacturer’s instructions.

### Wound healing assay

For kinetic migration analysis, cells were seeded to 80~90% confluency in 96-well plates (Essen Biosciences, MI). Wound areas were created using a 96-pin mechanical device (Incucyte® WoundMaker, Sartorius, NY) and washed twice with PBS to prevent reattachment of removed cells. Cells were treated with DOXA after scratch wound creation, and images of the wounds were automatically captured and registered every hour for 24 h using an IncuCyte^TM^ ZOOM^®^ Kinetic Imaging System. The Scratch Wound Cell Migration Software Module was utilized to analyze the relative wound density, assessing cell migration and closure over time.

### Serum biochemistry profiles for biomarkers of liver and renal injury

In order to assess the impact of DOXA on liver and kidney toxicity, blood samples were obtained from each animal upon sacrifice. Serum samples were then collected by centrifuging the blood at 3000 rpm for 20 min. The serum enzyme activities of aspartate aminotransferase (AST) and blood urea nitrogen (BUN) levels were measured with an AST and BUN assay kit according to the manufacturer’s protocol (Sigma-Aldrich). The concentrations of AST and BUN were determined by measuring the absorbance at 450 nm and 570 nm, respectively, using a Spectramax MAX 190 microplate reader (Molecular Devices).

### MMP-2, MMP-9 and VEGF ELISA assay

MMP-2, MMP-9 and VEGF levels in mouse serum were measured using ELISA kits (R&D Systems, Minneapolis, MN), following to the manufacturer’s instructions. The concentrations of MMP-2, MMP-9 or VEGF were determined by measuring the absorbance at 450 nm using a microplate reader.

### Public dataset source and bioinformatics analysis

Gene expression in normal and tumor tissues was analyzed using the publicly-available TCGA dataset. Data for survival analyses were downloaded from TCGA and GENT2 databases. Overall survival regression was analyzed with GraphPad Prism 9.0 software after categorization into high- and low-expression groups. Overall survival was analyzed up to 150 months, with *p*-values obtained through the *log-rank* test. Correlations of mRNA gene expression levels were analyzed using Pearson’s correlation coefficient (r).

### Statistical analysis

All data were analyzed using GraphPad Prism 9.0 statistical software (San Diego, CA). The results are presented as mean ± SEM of at least three independent experiments. Student's t-test, one-way or two-way ANOVA was performed as appropriate for data analysis. Significance between multiple experimental groups was determined using the Bonferroni post hoc test and defined at *p*<0.05.

## Results

### DOXA induces caspase activation and apoptosis in TNBC cells

**Fig. 1:**
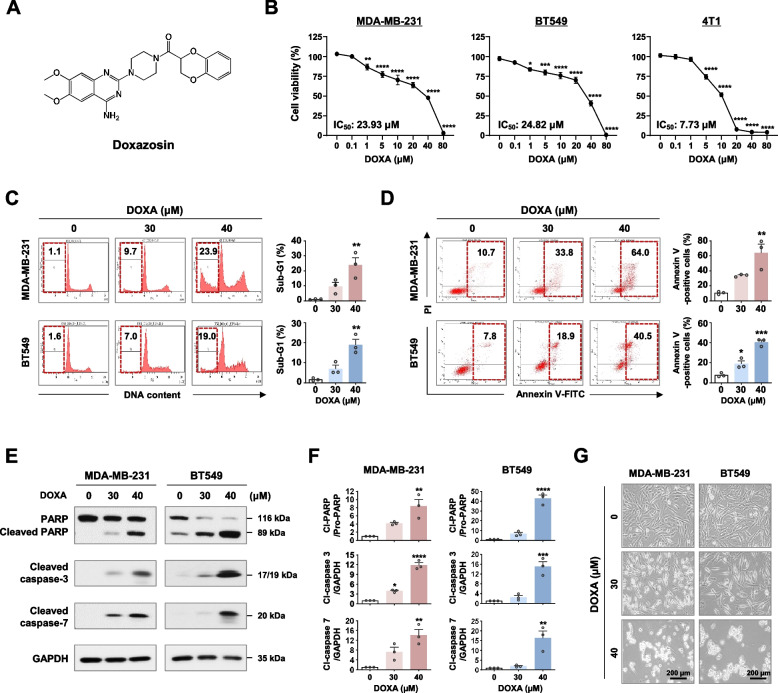
DOXA induces apoptosis in TNBC cells. **A** Chemical structure of doxazosin (DOXA). **B** Effect of DOXA on cell viability in TNBC cells. MDA-MB-231, BT549 and 4T1 cells were treated with various concentrations of DOXA (0.1-80 μM) or control vehicle (DMSO) for 48 h. Cell viability and IC_50_ values were determined by MTS assay (*p<0.05). **C**, **D** MDA-MB-231 and BT549 cells were treated with DOXA (0-40 μM, 48 h). The sub-G1 population (**C**, **p<0.01) and annexin V-positive cells (**D**, *p<0.05) were quantified using flow cytometry. **E** Immunoblot analyses of PARP, cleaved caspase-3 and -7 expression in MDA-MB-231 and BT549 cells following exposure to DOXA (0-40 μM, 48 h). GAPDH was used as an internal control. **F** Quantitative graphs represent the ratio of protein content (*p<0.05). **G** Morphological changes in MDA-MB-231 and BT549 cells after treatment with DOXA (0-40 μM, 48 h). Original magnification: × 200

To evaluate the anti-proliferative effects of DOXA in TNBC, MDA-MB-231, BT549, and mouse 4T1 cell lines were treated with varying concentrations of DOXA for 48 h. The MTS assay revealed a significant reduction in cell viability in response to DOXA treatment (0.1–80 µM) in a dose-dependent manner (*p*<0.05, Fig. 1B). The calculated IC_50_ values for DOXA were 23.93 μM, 24.82 μM and 7.73 μM in MDA-MB-231, BT549 and 4T1, respectively. Based on the IC_50_ value of DOXA, we selected a dose range of 10-40 μM for further in vitro experiments. DOXA treatment (30–40 μM, 48 h) effectively evoked apoptosis in MDA-MB-231 and BT549 cells, as evidenced by a notable accumulation of the sub-G1 population (*p*<0.01, Fig. 1C) and a substantial increase in both early and late apoptotic cell populations (*p*<0.05, Fig. 1D). This phenomenon was accompanied by typical apoptotic events including caspase-3 and -7 activation and increased PARP cleavage (*p*<0.05, Fig. 1E, F), as well as cell morphological changes with apoptotic bodies (Fig. 1G). These events were also consistently observed in 4T1 cells following exposure to DOXA (10–20 μM, 48 h) (*p*<0.01, Supplementary Figs. S1 and S2). We also evaluated the anti-proliferative effect of c-MET inhibitors (crizotinib, capmatinib and tepotinib) and EGFR inhibitors (lazertinib and osimertinib) in MDA-MB-231, BT549 and 4T1 cells. The TNBC cell lines were sensitive to both the c-MET inhibitors and the EGFR inhibitors (0.001~100 µM, 48 h; Supplementary Table S2). Of particular note, the human bispecific antibody amivantamab (50~1000 µg, 72 h) significantly suppressed cell viability (Supplementary Fig. S3) in agreement with previous observations in NSCLC [21]. These results suggest that dual blockade of c-MET/EGFR could be an effective strategy to suppress TNBC.

### DOXA suppresses c-MET and its downstream signaling pathways

**Fig. 2:**
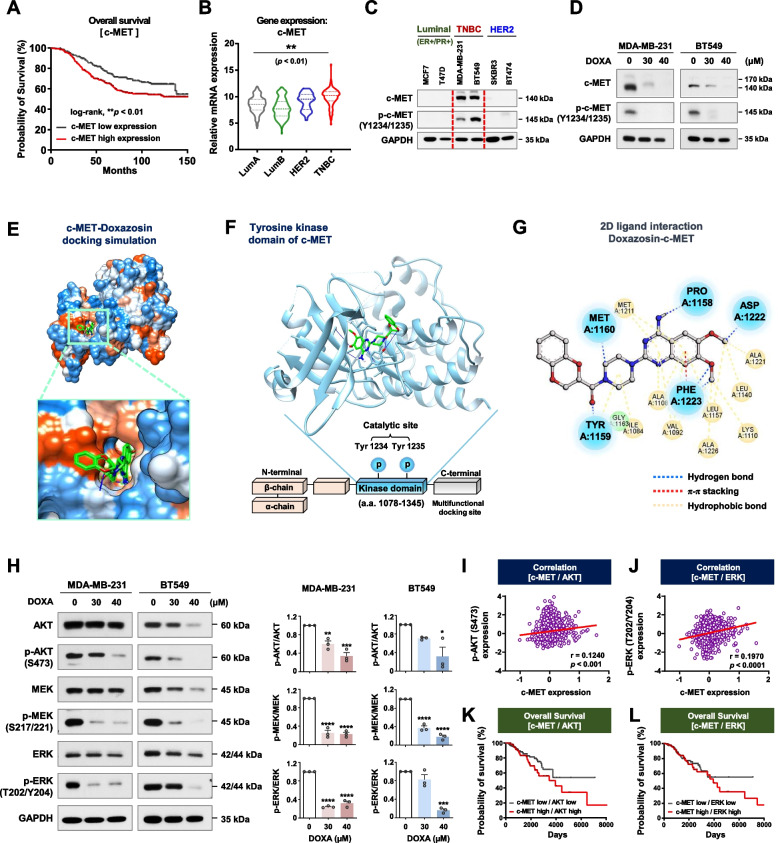
DOXA targets the c-MET signaling pathway. **A** Kaplan-Meier analysis for overall survival in breast cancer patients stratified by c-MET gene expression using data from the GENT2 database [log-rank; *p*=0.0076, c-MET-high (*n*=267) and c-MET-low (*n*=228)]. **B** Comparison of c-MET mRNA expression in subtypes of breast cancer patients by TCGA dataset analysis [(**p<0.01, Luminal A (*n*=491), Luminal B (*n*=210), HER2 (*n*=76) and TNBC (*n*=165)]. **C** Immunoblot analyses for c-MET and phospho-c-MET (Y1234/1235) expression in six breast cancer cell lines. **D** Changes in the expression of c-MET and phospho-c-MET in MDA-MB-231 and BT549 cells following exposure to DOXA (0-40 μM, 48 h). **E**-**G** *In silico* molecular docking analysis between DOXA and c-MET (PDB: 6SD9). **E** Surface map of lipophilic/hydrophilic properties in the active binding site of c-MET (red: hydrophobic, blue: hydrophilic). **F** Binding pose of DOXA (green stick model) in the tyrosine kinase domain of c-MET (blue ribbon). **G** 2D diagram analysis of intermolecular interactions between DOXA and c-MET. Key amino acid residues within the binding pocket are displayed in ball-and-stick format. Hydrogen bonds (< 4.0 Å), π-π stacking and hydrophobic bonds are represented as blue, red and yellow dashed lines, respectively. **H** Immunoblot analyses for AKT, phospho-AKT (S473), MEK, phospho-MEK (S217/221), ERK and phospho-ERK (T202/Y204) expression in MDA-MB-231 and BT549 cells following exposure to DOXA (0-40 μM, 48 h). Quantitative graphs represent the ratio of phosphorylated-protein/total-protein expression (right panel, *p<0.05). **I**, **J** Correlation between c-MET and either phospho-AKT (S473) (**I**, ***p<0.001) or phospho-ERK (T202/Y204) (**J**, ****p<0.0001) protein expression in breast cancer patients. The co-expression score between two genes was calculated by Pearson’s correlation coefficient (r). **K**, **L** Overall survival rates for breast cancer patients with high and low protein expression levels stratified by c-MET and AKT (**K**) and between c-MET and ERK (**L**) expression (TCGA cohort)

Dysregulated c-MET activation drives cancer cells to survive, proliferate, and metastasize, and is linked to undesirable clinical outcomes [5, 22]. GENT2 dataset analysis revealed that overall survival was significantly poorer in breast cancer patients with high c-MET expression (Log-rank, *p*<0.01, Fig. 2A). According to the TCGA database, c-MET mRNA expression levels in TNBC patients are higher than in other subtypes of breast cancer (*p*<0.01, Fig. 2B). In immunoblot analysis of a panel of breast cancer cell lines, we observed that the protein content and phosphorylation status of c-MET were relatively higher in TNBC when compared to luminal or HER2-positive subtypes (Fig. 2C).

Following exposure to DOXA (30–40 μM, 48 h), c-MET was dose-dependently degraded and a significant decrease in phosphorylation of residues Tyr1234/1235 in the tyrosine kinase domain was observed in MDA-MB-231 and BT549 cells (Fig. 2D; *p*<0.001, Supplementary Fig. S4). We performed a molecular docking simulation to clarify whether this phenomenon was due to the direct binding of DOXA to c-MET (Fig. 2E). Docking studies using the crystal structure of c-MET (PDB: 6SD9) revealed that DOXA comfortably fits into the catalytic site of the tyrosine kinase domain, located in the C-terminal region of its β-chain (Fig. 2F). Their interaction is extensively stabilized by six hydrogen bonds with amino acid residues Pro1158, Tyr1159, Met1160, Asp1222 and Phe1223 of c-MET in the dimethoxyquinazoline and benzodioxin groups of DOXA (Fig. 2G). In addition, one π-stacking interaction was formed between the active residue Phe1223 and a quinazoline ring of DOXA. It is noteworthy that DOXA forms hydrogen bonds with key residues within the ATP-binding site of c-MET, including Pro1158, Tyr1159, and Met1160. The interaction is similar to that reported for crizotinib, an FDA-approved type I c-MET inhibitor [23]. The molecular docking analysis of DOXA with c-MET using the DockThor server predicted a high binding affinity (-9.226 kcal/mol) and interaction energy (-37.413 kcal/mol) (Supplementary Table S3). The predicted binding affinity values of the c-MET inhibitors (crizotinib, capmatinib, and tepotinib) with c-MET (6SD9) were -9.179, -9.744, and -9.793 (kcal/mol), respectively. It is noteworthy that the predicted binding affinity of DOXA and c-MET was higher than crizotinib (Supplementary Table S4). These results provide insights into the mechanism of action of DOXA.

We next examined whether the blockade of c-MET by DOXA attenuates AKT and MEK/ERK activation. Treatment with DOXA (30–40 μM, 48 h) resulted in a significant reduction in the expression levels and phosphorylation of AKT, MEK, and ERK in TNBC (*p*<0.05, Fig. 2H). TCGA dataset analysis revealed a statistically significant correlation in protein expression between c-MET and phospho-AKT as well as phospho-ERK (*p*<0.001, Fig. 2I, J), while breast cancer patients with high expression levels of c-MET/AKT and c-MET/ERK. (Fig. 2K, L) had lower overall survival.

### DOXA targets EGFR and downregulates P-glycoprotein

**Fig. 3:**
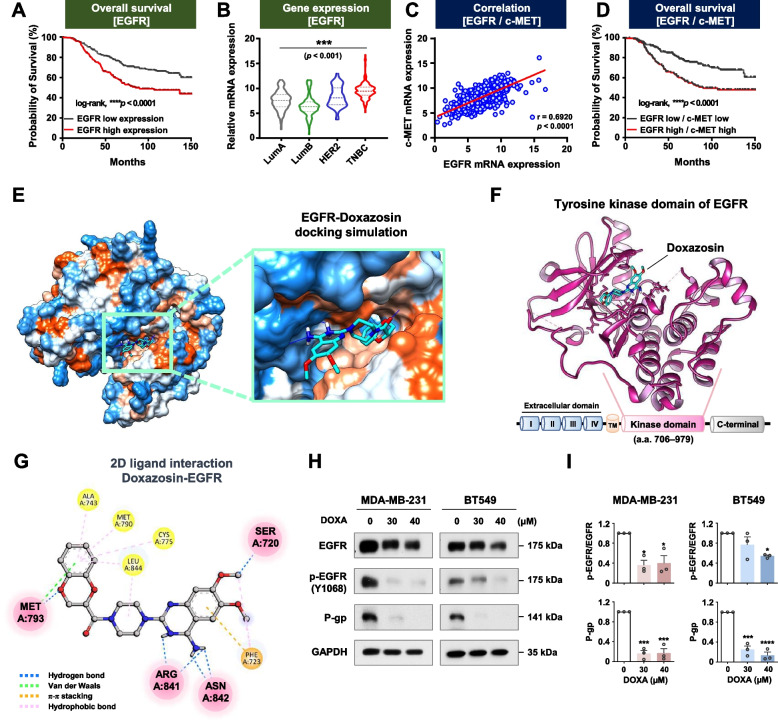
DOXA impedes EGFR activation via direct binding to its tyrosine kinase domain. **A** Overall survival depicted by Kaplan-Meier curve of breast cancer patients with high and low EGFR gene expression following GENT2 dataset analysis [log-rank; ****p<0.0001, EGFR-high (*n*=194) and EGFR-low (*n*=298)]. **B** Analysis of mRNA expression of EGFR in subtypes of breast cancer patients using TCGA cohort data [***p<0.001, Luminal A (*n*=491), Luminal B (*n*=210) HER2 (*n*=76) and TNBC (*n*=165)]. **C** Correlation of mRNA expression between EGFR and c-MET in breast cancer patients (****p<0.0001). **D** Overall survival for breast cancer patients with high or low mRNA levels between EGFR and c-MET in the GENT2 cohort (****p<0.0001). **E**-**G** Molecular docking simulation between DOXA and EGFR (PDB: 5CAP). **E** Lipophilicity surface map of the active binding site of EGFR. **F** Docked position of DOXA (blue stick model) in the tyrosine kinase domain of EGFR (pink ribbon). **G** Intermolecular interactions depicted by 2D diagram between DOXA and EGFR. Hydrogen bonds (< 4.0 Å), π-π stacking and hydrophobic bonds are represented as blue, orange and pink dashed lines, respectively. **H** Immunoblot analyses of EGFR, phospho-EGFR (Y1068), and P-glycoprotein expression in MDA-MB-231 and BT549 cells following exposure to DOXA (0-40 μM, 48 h). **I** Quantitative graphs represent the ratio of phospho-EGFR/total-EGFR and P-glycoprotein/GAPDH (*p<0.05)

Simultaneous overexpression of c-MET and EGFR in TNBC is correlated with more aggressive clinicopathological features and poorer clinical outcomes [24]. In the GENT2 and TCGA cohort analysis, EGFR mRNA expression was highest in TNBC, and breast cancer patients in the high expression group showed a notably lower probability of overall survival (*p*<0.001, Fig. 3A, B). A significant correlation was found between mRNA abundance of c-MET and EGFR in breast cancer patients, and the concurrent overexpression of these genes was associated with comparatively poorer overall survival outcomes (*p*<0.0001, Fig. 3C, D).

To evaluate whether the quinazoline-based DOXA directly interacts with EGFR, a molecular docking simulation using the established EGFR crystal structure (PDB: 5CAP) was conducted. DOXA is tightly anchored between the hydrophobic and hydrophilic regions within the active binding site of EGFR (Fig. 3E). The predicted docking model showed that DOXA fits into the ATP-binding pocket of tyrosine kinase in EGFR (Fig. 3F). This interaction is stably formed by six hydrogen bonds, one π-stacking and several hydrophobic interactions (Fig. 3G). The predicted binding affinity and interaction energy were calculated at -9.004 kcal/mol and -37.328 kcal/mol (Supplementary Table S3). Osimertinib and lazertinib are third-generation EGFR-TKIs that selectively and irreversibly inhibit both EGFR-sensitizing and T790M mutations in NSCLC [25, 26]. The calculated binding affinity values for osimertinib and lazertinib with EGFR (5CAP) were -8.947 and -9.743 kcal/mol, respectively. Notably, the binding affinity of DOXA with EGFR was higher than that of osimertinib (Supplementary Table S5). The interaction between DOXA and EGFR resulted in a marked downregulation of expression and phosphorylation of EGFR protein in TNBC cells after treatment with DOXA (30–40 μM, 48 h) (*p*<0.05, Fig. 3H, I). TKIs including crizotinib and brigatinib frequently elicit drug resistance via overexpression of MDR protein members such as P-glycoprotein (or MDR1) [27, 28]. In contrast, exposure to DOXA resulted in a dramatic decrease in mRNA abundance and protein content of P-glycoprotein in TNBC cells (Fig. 3H, I and Supplementary Fig. S5), suggesting the potential to overcome a major obstacle in chemoresistance.

### DOXA impairs CSC-like properties by disrupting the CD44-EGFR axis

**Fig. 4:**
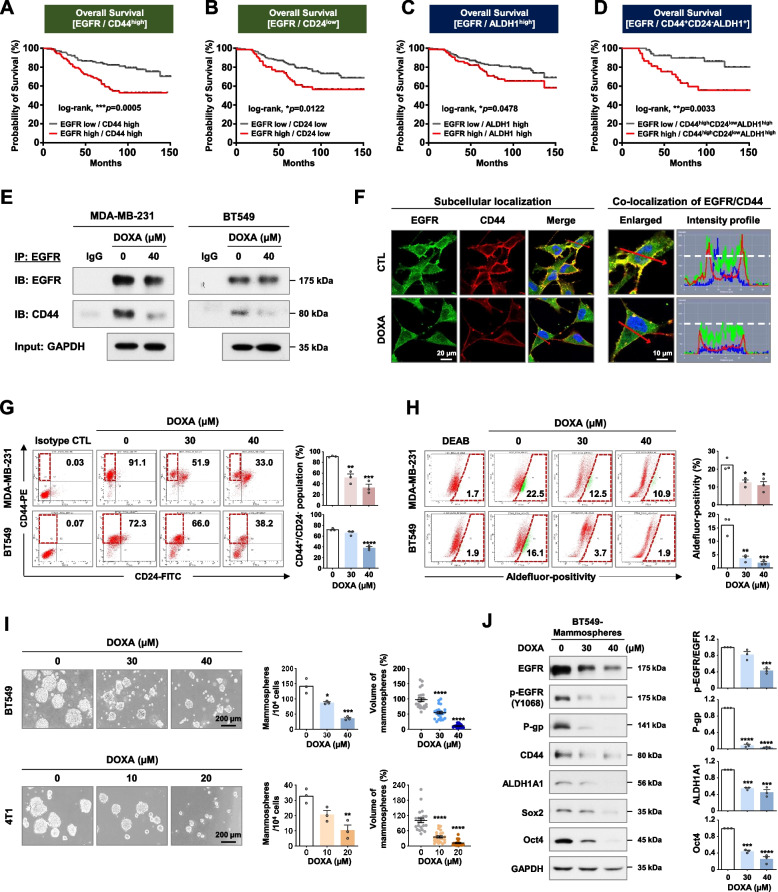
DOXA impairs CSC-like properties by disrupting the EGFR/CD44 axis. **A-D** Overall survival depicted by Kaplan-Meier curve is shown **A** between EGFR and CD44-high [log-rank; *p*=0.0005, EGFR-high/CD44-high (*n*=92) and EGFR-low/CD44-high (*n*=111)], **B** between EGFR and CD24-low [log-rank; *p*=0.0122, EGFR-high/CD24-low (*n*=66) and EGFR-low/CD24-low (*n*=140)], **C** between EGFR and ALDH1-high [log-rank; *p*=0.0478, EGFR-high/ALDH1-high (*n*=85) and EGFR-low/ALDH1-high (*n*=156)], and **D** between EGFR and CD44-high/CD24-low/ALDH1-high [log-rank; *p*=0.0033, EGFR-high (*n*=37) or EGFR-low (*n*=53)] in the GENT2 dataset. **E** MDA-MB-231 and BT549 cells were treated with DOXA (40 μM) for 24 h. Cell lysates were immunoprecipitated (IP) with anti-EGFR antibody and analyzed by immunoblotting (IB) with CD44 antibody. IgG, normal mouse immunoglobulin G. **F** MDA-MB-231 cells were immunostained for CD44 and EGFR with DAPI following exposure to DOXA (40 μM, 24 h). The colocalization between CD44 and EGFR was analyzed by confocal microscopy using the profile intensity tool (red arrows). The horizontal white line indicates 150 intensity units (y-axis). **G**,** H** Influence of DOXA on CSC-like characteristics in TNBC cells. Cells were treated with DOXA (0-40 µM, 48 h). CD44^high^/CD24^low^ populations (**G**, **p<0.01) and Aldefluor-positivity (**H**, *p<0.05) were determined by flow cytometry. **I** BT549 (0.7×10^4^ cells/ml) and 4T1 (0.2×10^4^ cells/ml) were cultured in serum-free suspension conditions in the presence or absence of DOXA for 5 days. The number and volume of mammospheres was quantified by optical microscopy (*p<0.05). **J** Changes in EGFR, phospho-EGFR (Y1068), P-glycoprotein, CD44, ALDH1A1, Sox2, and Oct4 expression in BT549 mammospheres following exposure to DOXA (0-40 μM, 5 days)

CD44 is a surface marker of CSCs and has been reported to act as a co-receptor for EGFR that activates downstream signaling in breast cancer [29]. To explore the correlation between EGFR and CD44, we evaluated the prognostic value of these proteins on overall survival according to mRNA expression levels in breast cancer patients. In the GENT2 dataset, patients with high CD44 mRNA expression had a poorer survival rate in the high EGFR expression group (Log-rank, *p*=0.0005, Fig. 4A). Similarly, high EGFR expression was associated with relatively worse overall survival in patients with other CSC-like characteristics including low CD24 (*p*=0.0122, Fig. 4B), high ALDH1 (*p*=0.0478, Fig. 4C) and the CD44^high^/CD24^low^/ALDH1^high^ phenotype (*p*=0.0033, Fig. 4D). In addition, immunoprecipitation assays with anti-EGFR antibody revealed that DOXA (40 µM, 24 h) inhibits the interaction between EGFR and CD44 in both MDA-MB-231 and BT549 cells (Fig. 4E and Supplementary Fig. S6). Double-fluorescence immunochemistry further revealed that EGFR and CD44 are predominantly distributed and co-localized in the cytoplasmic membrane, highlighted by intensively overlapping fluorescent signal, which was markedly diminished in the presence of DOXA (40 µM, 24 h) in MDA-MB-231 cells (Fig. 4F). Our findings indicate that DOXA has the potential to disrupt the EGFR/CD44 axis, leading to the impairment of CSC-like properties.

We next sought to examine whether DOXA impairs CSC-like traits. The CD44^high^/CD24^low^ subpopulation (*p*<0.01, Fig. 4G) and ALDH1 activity (*p*<0.05, Fig. 4H) in MDA-MB-231 and BT549 cells were significantly decreased following exposure to DOXA (30–40 µM, 48 h). Consistent with these results, DOXA (10–20 µM, 48 h) also suppressed the murine mammary stem-like characteristics of CD49f^high^/CD24^high^ and ALDH1 activity in 4T1 cells (*p*<0.05, Supplementary Fig. S7).

We further confirmed the effect of DOXA on CSC-like behavior using a mammosphere assay in vitro. DOXA treatment significantly diminished the mammosphere-forming ability, as indicated by a pronounced reduction in both the number and volume of mammospheres derived from BT549 and 4T1 cells (*p*<0.05, Fig. 4I). An immunoblot analysis revealed that DOXA eradicates CSC-like characteristics in mammospheres via reduced levels of CD44, ALDH1A1, Sox2 and Oct4. The expression of EGFR and phospho-EGFR was also downregulated by DOXA challenge, concomitant with a significant reduction in P-glycoprotein protein content (*p*<0.001, Fig. 4J).

### DOXA impedes lung colonization of CSC-like subpopulations via suppression of STAT3 signaling

**Fig. 5:**
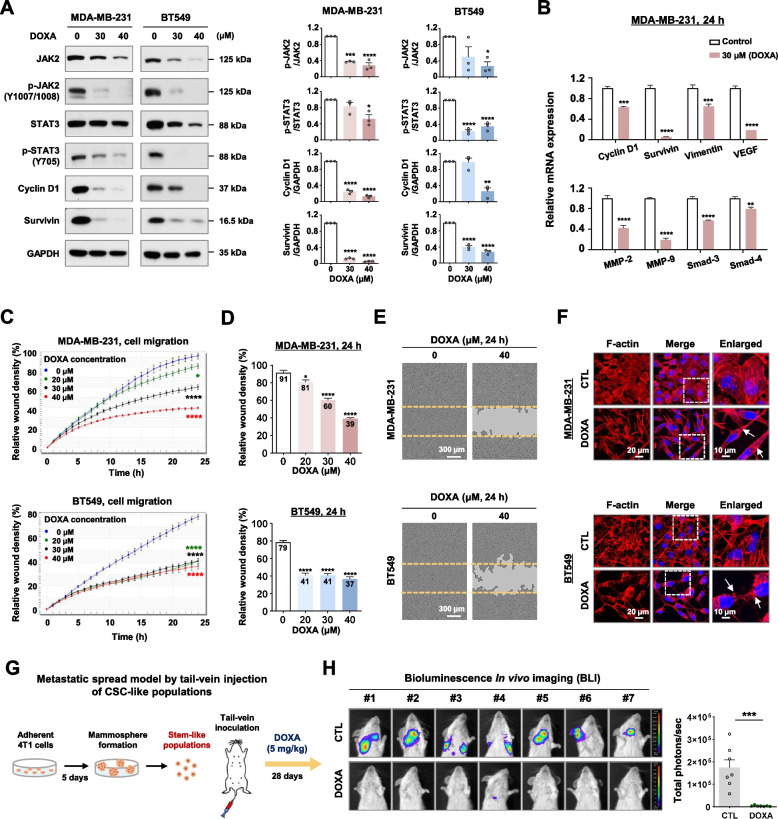
DOXA impairs metastatic ability by suppressing JAK/STAT3 signaling. **A** Immunoblot analyses of JAK2, phospho-JAK2 (Y1007/1008), STAT3, phospho-STAT3 (Y705), cyclin D1, and survivin expression in MDA-MB-231 and BT549 cells after treatment of DOXA (0-40 μM, 48 h). Quantitative graphs represent the ratio of phosphorylated-protein/total-protein or the protein content relative to GAPDH expression (*p<0.05). **B** Relative mRNA expression of cyclin D1, survivin, vimentin, MMP-2, MMP-9, VEGF, smad-3 and smad-4 was analyzed by quantitative RT-PCR in MDA-MB-231 cells after treatment with DOXA (0-30 μM, 24 h) (**p<0.01). **C-E** Impact of DOXA on cell migration. **C** Following exposure to DOXA (0-40 μM, 24 h) in MDA-MB-231 and BT549 cells, kinetic analysis of cell migration was determined and quantified for the indicated time duration (*p<0.05). **D** Relative wound density (%) at 24 h (*p<0.05). **E** Representative images of wound closure by cell migration at 0 and 24 h after treatment with DOXA (40 μM). The yellow dotted line indicates the edge of the scratched wound. **F** MDA-MB-231 and BT549 cells were immunostained with F-actin (1:100, Texas Red=X phalloidin) with DAPI (blue) after treatment with DOXA (0-40 μM) for 24 h. White arrows indicate the disruption of actin filaments. **G**,** H** Effect of DOXA on lung colonization using an experimental metastasis model in vivo. **G** 1×10^5^ cells from 4T1 mammosphere cultures were inoculated into the tail vein of BALB/c female mice and immediately injected intravenously with DOXA (5 mg/kg) or solvent control. **H** The degree of lung colonization from control or DOXA-treated mice was evaluated using luminescence signal intensity (total photons/second, ***p<0.001, *n*=7)

Aberrant activation of JAK/STAT3 signaling, downstream of c-MET and EGFR, is observed in TNBC and contributes to cell survival, invasion, migration, angiogenesis, and metastasis [30]. Exposure to DOXA (30–40 µM, 48h) significantly suppressed the activation of JAK2 and STAT3 in TNBC cells and subsequently downregulated the downstream targets cyclin D1 and survivin (*p*<0.05, Fig. 5A). The mRNA expression of STAT3 downstream targets including cyclin D1, survivin, vimentin, VEGF, MMP-2 and MMP-9 as well as epithelial-mesenchymal transition (EMT)-inducing transcriptional factors Smad-3 and Smad-4 were consistently repressed after treatment with DOXA (30 µM, 24h) in MDA-MB-231 cells (*p*<0.01, Fig. 5B). Kinetic analysis revealed that DOXA dose-dependently reduced migratory ability in both MDA-MB-231 and BT549 cells, as well as in 4T1 cells (*p*<0.05, Fig. 5C–E; *p*<0.05, Supplementary Fig. S8), accompanied by the disruption of F-actin filament (Fig. 5F).

CSCs become enriched during mammosphere formation partly due to their ability to survive under anchorage-independent conditions [31]. We further investigated the effect of DOXA on the dissemination and lung colonization of CSCs using an in vivo experimental metastasis model. 1×10^5^ cells dissociated from 4T1 mammospheres with enriched CSC-properties were inoculated into the tail vein of BALB/c female mice followed by a single intravenous injection of 5 mg/kg of DOXA (Fig. 5G). After 28 days, in vivo BLI analysis revealed a dramatic impediment in the luminescence signal intensity representing lung colonization by 4T1 spheroid cells in the group treated with DOXA (*p*<0.001, Fig. 5H).

### DOXA inhibits tumor growth in CSC-enriched TNBC allografts in vivo

**Fig. 6:**
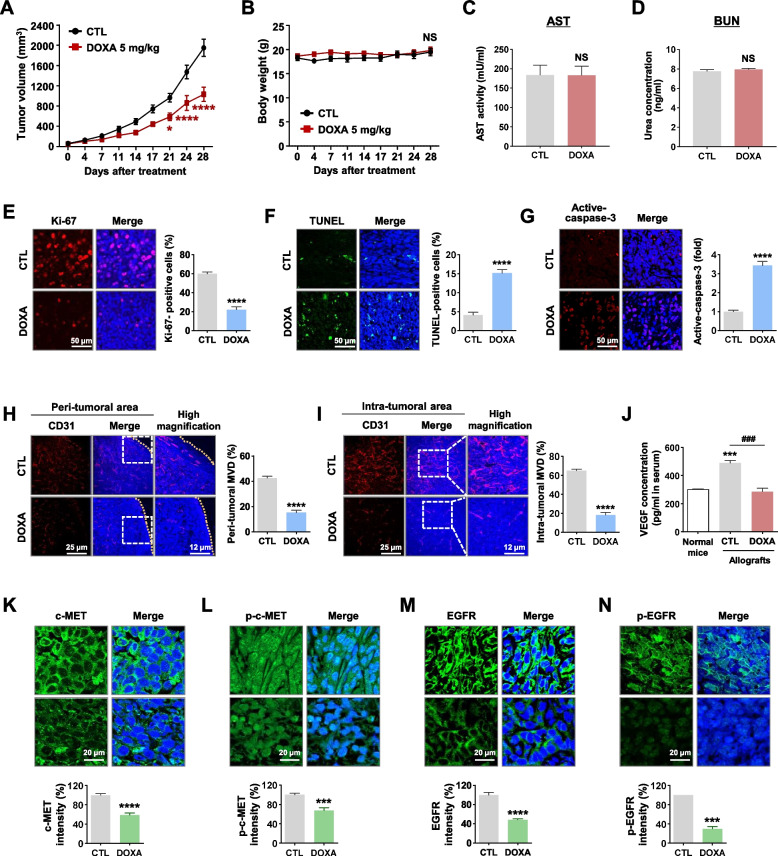
DOXA retards tumor growth in CSC-enriched 4T1 allografts. **A**, **B** Effects of DOXA on tumor growth in vivo. 1×10^5^ cells from 4T1 mammospheres cultures were orthotopically injected into the fourth mammary gland of mice. Following exposure to DOXA (5 mg/kg, every other day) or control vehicle in allografted mice, tumor growth (**A**, *p<0.05, *n*=5) and body weight (**B**, NS, not significant) were evaluated. **C**, **D** Influence of DOXA on serum biochemical parameters of hepatic and renal toxicity. Blood biochemical analyses indicated there were no significant changes in serum AST or BUN (NS; *n*=5). **E-G** Impact of DOXA on Ki-67 expression and apoptosis in vivo*.* Immunostaining of tissue sections was performed using Ki-67 (**E**) with DAPI and the quantitative graph represents the percentage of Ki-67-positive cells (****p<0.0001). DOXA-induced apoptosis was determined by TUNEL-positive cells (**F**, ****p<0.0001) and high expression of cleaved-caspase-3 (**G**, ****p<0.0001). **H**,** I** Influence of DOXA on tumor angiogenesis. The microvessel density (MVD, the number of CD31-positive microvessels) was quantified in the peri-tumoral (**H**, ****p<0.0001) and intra-tumoral areas (**I**, ****p<0.0001). Original magnification: × 200. **J** Changes in serum levels of VEGF in allografted mice following DOXA administration. Normal mouse serum was used as a negative control (###*p*<0.001, control vs. DOXA-treated allografts; ***p<0.001, normal mice vs. control allografts). **K-N** Immunohistochemical analysis for c-MET (**K**), phospho-c-MET (Y1234/1235, **L**), EGFR (**M**), and phospho-EGFR (Y1068, **N**). Fluorescence intensities of both total and phosphorylated c-MET and EGFR expressions were quantified (***p<0.001)

We further investigated whether DOXA elicits in vivo antitumor activity to confirm the physiological relevance of the in vitro findings. To achieve this, we selected for intraperitoneal (IP) administration, which allows for rapid absorption due to a greater surface area compared to oral administration. This approach minimizes the potential for degradation or modification influenced by the gastrointestinal tract [32]. In an orthotopic allograft model derived from CSC-enriched 4T1 spheroid cells (1×10^5^), administration of DOXA (5 mg/kg, every other day, 28 days) resulted in significant attenuation of tumor growth, without significant bodyweight change (*p*<0.05, Fig. 6A, B). Furthermore, DOXA did not appear to impact hepatic and renal function, evidenced by stable serum levels of AST and BUN (NS, not significant, Fig. 6C, D). Immunofluorescence staining for Ki-67, a proliferation indicator, revealed that the number of Ki-67-positive cells was markedly reduced in the DOXA-treated allograft tumors (*p*<0.0001, Fig. 6E). In addition, DOXA administration led to a significant increase in the apoptotic index as determined by TUNEL-positivity and caspase-3 activation in allograft tumors (*p*<0.0001, Fig. 6F, G). To investigate the effect of DOXA on angiogenesis, immunostaining for cluster of differentiation 31 (CD31) was performed to measure microvessel density (MVD). The number of CD31-positive microvessels was markedly reduced in both intra- and peri-tumoral areas in the DOXA-treated group (*p*<0.0001, Fig. 6H, I), accompanied by decreased levels of VEGF in serum (*p*<0.001, Fig. 6J). As supported by the molecular docking studies and in vitro observations, DOXA exhibited potent antitumor activity in TNBC allograft tumors via direct targeting of both c-MET and EGFR activation in vivo (*p*<0.001, Fig. 6K-N).

### DOXA suppresses distant metastasis by targeting CSC-like traits in vivo

**Fig. 7:**
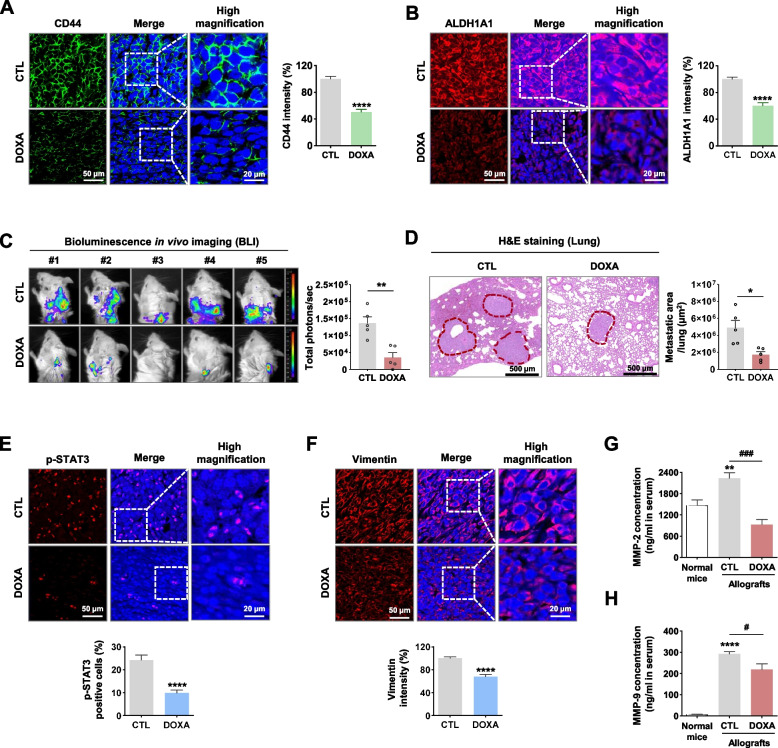
DOXA suppresses TNBC metastasis. **A, B** Effect of DOXA on the expression of CSC markers CD44 and ALDH1A1 in allograft tumors derived from 4T1 mammospheres*.* Fluorescence intensities of CD44 (**A**, ****p<0.0001) and ALDH1A1 (**B**, ****p<0.0001) were quantified. **C** Representative BLI of metastasis in the CTL- and DOXA-treated group. DOXA administration resulted in a marked decrease in bioluminescent signal intensity (total photons/second, **p<0.01, *n*=5). **D** Hematoxylin and eosin (H&E) staining in lung sections from CTL- and DOXA-treated mice. The red dotted areas indicate metastatic lesions in lungs. The number of tumor nodules in lungs was quantified (*p<0.05, *n*=5). **E**, **F** Immunohistochemical analysis for phospho-STAT3 (Y705) and vimentin in allograft tumors. phospho-STAT3-positive cells were counted (**E**, ****p<0.0001), and fluorescence intensities of vimentin expression were quantified (**F**, ****p<0.0001). **G**, **H** Impact of DOXA on serum levels of MMP-2 and MMP-9 in vivo. MMP-2 and MMP-9 expression was determined by ELISA assay in serum collected from the CTL- and DOXA-treated mice (**p<0.01, normal mice vs. control allografts; #*p*<0.05, control allografts vs. DOXA-treated allografts)

TNBCs with higher CSC-like populations exhibit a more aggressive metastatic phenotype correlating with persistent activation of STAT3 [33]. Significant decreases in the CSC markers CD44, ALDH1A1 and CD49f were observed in the DOXA-treated allograft tumors (*p*<0.0001, Fig. 7A, B; *p*<0.0001, Supplementary Fig. S9). Based on these findings, we further examined whether DOXA influences metastasis from the primary tumor to distant organs in 4T1 mammosphere allografts. Although the metastasis to distant organs occurred entirely in control allograft mice, DOXA administration resulted in significant reductions in the bioluminescence intensity and histopathological lesions for distant areas (*p*<0.05, Fig. 7C, D). In agreement with the in vitro findings, the DOXA-treated allograft tumors exhibited a notable decrease in the number of phospho-STAT3-positive cells. This response was accompanied by subsequent reductions in the expression levels of vimentin and the serum concentrations of MMP-2 and MMP-9 (*p*<0.05, Fig. 7E-H). These findings suggest that DOXA-induced anti-metastatic activity is correlated with the eradication of CSC-like traits and disruption of STAT3 signaling.

## Discussion

Drug repositioning reduces the risk of clinical development failures and can benefit from established data on pharmacology, dosing, and potential toxicity compared to *de novo* drugs. Clinical pharmacokinetic profiles revealed that DOXA exhibits rapid absorption, achieving peak plasma levels around 3 hours after oral administration, with an oral bioavailability of 62%-69% and a half-life of 10-12 hours when administered as a single dose [34, 35]. In healthy individuals, a single oral dose of 1 mg DOXA resulted in peak plasma concentrations of 7.6 ng/ml at 3.6 hours, while hypertensive patients achieved 76 ng/ml within 2-3 hours with an 8 mg dose [34]. A pharmacokinetic study in non-fasted rodents has reported that after a single oral administration of 8 mg/kg DOXA, the plasma concentration was at 200 ng/ml at 2 h [36]. Therefore, we estimate that when 5 mg/kg DOXA used in our in vivo experiments was orally administered to mice as a single dose, peak plasma concentrations could be achieved at approximately 125 to 187.5 ng/ml. This is expected to be similar to the mean peak plasma concentrations of 150 ng/ml achieved with a single administration of 16 mg DOXA in hypertensive patients.

In preclinical studies, oral administration of DOXA in mice results in an LD50 exceeding 1000 mg/kg. No toxicity has been observed in canines even after oral administration of the maximum dose of 16 mg/kg/day for three months [37]. A broad therapeutic index is particularly important in cancer treatment, as many patients receive combination therapies. Our in vivo findings show that treatment of DOXA (5 mg/kg, every other day) for four weeks significantly suppresses tumor growth and metastasis without significant impact on renal or hepatic function in mice.

Evidence suggests that simultaneous overexpression of c-MET and EGFR in TNBC exacerbates anticancer drug resistance leading to metastasis [38]. High expression levels of both EGFR and c-MET in a TCGA data cohort significantly correlate with unfavorable overall survival in breast cancer patients. Crizotinib (PF-02341066) is a type I c-MET inhibitor for metastatic NSCLC and is currently in clinical trials for the treatment of metastatic breast cancer [22]. In our docking studies, DOXA forms hydrogen bonds with Pro1158, Tyr1159, and Met1160, which constitute the ATP binding sites of c-MET, with the interaction being similar to crizotinib [23]. DOXA also interacts with the tyrosine kinase domain of EGFR, specifically through hydrogen bonds and pi-pi stacking involving amino acid residues Arg841 and Phe723. The crosstalk between EGFR and c-MET enhances chemoresistance by activating downstream survival pathways such as PI3K/AKT, MAPK, and JAK/STAT3 [39].

CD44 is a multifunctional glycoprotein that plays a pivotal role in various facets of metastasis by mediating cell-cell adhesion, cell-extracellular matrix (ECM) interactions, and cytoskeletal networks [40]. It also promotes resistance to anoikis, enabling CSCs to evade cell death and generate metastatic colonies in secondary organs [41]. Our immunoprecipitation assay showed that DOXA treatment induces a notable decrease in the interaction between EGFR and CD44 in TNBC cells, thus supporting its potential to disrupt the EGFR/CD44 axis and effectively attenuate CSC-like properties. The inhibitory effect of DOXA on CSC-like properties was concomitant with the suppression of CD44^high^/CD24^low^ subpopulations and ALDH1 activity. ALDH1 is an enzyme that plays a vital role in detoxifying both endogenous and exogenous aldehydes. Its high detoxification and antioxidant activities, including the scavenging of reactive oxygen species (ROS), contribute to the protection of CSCs, thereby impeding the efficacy of chemotherapy [42].

P-glycoprotein, also known as MDR1, is markedly upregulated in TNBCs and facilitates the efflux of chemotherapeutic agents such as doxorubicin and paclitaxel to enable cancer cell survival [27, 43]. Our previous observations indicate that P-glycoprotein is predominantly expressed in TNBC, especially in the mesenchymal stem-like subtype, which is associated with lower survival and higher heterogeneity. Notably, P-glycoprotein is upregulated in stem-like populations including ALDH1-positive TNBC cells and mammospheres [28]. In the present study, DOXA effectively downregulated P-glycoprotein in CSC-enriched mammospheres.

STAT3 is a downstream effector of c-MET/EGFR and is activated in CSCs and primarily expressed in the invasive tumor margin [22, 44]. The transcription factor facilitates EMT, tumor angiogenesis and chemoresistance by upregulating the expression of key intermediaries such as VEGF, vimentin, HIF-1α and P-glycoprotein [20, 33, 45]. The downregulation of P-glycoprotein was attributed to disruption of STAT3 by DOXA challenge. Moreover, STAT3 promotes the expression of MMP-2 and MMP-9, crucial enzymes that participate in proteolytic degradation and reorganization of the ECM and basement membranes during the process of angiogenesis and metastasis [45]. Kinetic migration assays revealed that DOXA exerts inhibitory effects on TNBC cell migration in vitro. This phenomenon is likely attributed to the downregulation of vimentin, a pivotal factor involved in EMT and the acquisition of cellular motility [46]. Furthermore, administration of DOXA effectively attenuated elevated levels of VEGF, MMP-2, and MMP-9 observed in circulating blood during the progression of metastasis in allografts.

## Conclusion

Our findings shed light on the significant anti-tumor and anti-metastatic effects of DOXA, a promising drug candidate for repurposing to treat metastatic TNBC. Dual inhibition of c-MET and EGFR has been receiving increasing attention, with the development of amivantamab by Janssen Pharmaceuticals currently in phase 3 trials for the treatment of NSCLC [47]. Simultaneous overexpression of c-MET and EGFR in TNBC is strongly correlated with drug resistance and unfavorable clinical outcomes. While new generation c-MET and EGFR inhibitors show promise, their high cost limits accessibility to patients globally.

**Fig. 8:**
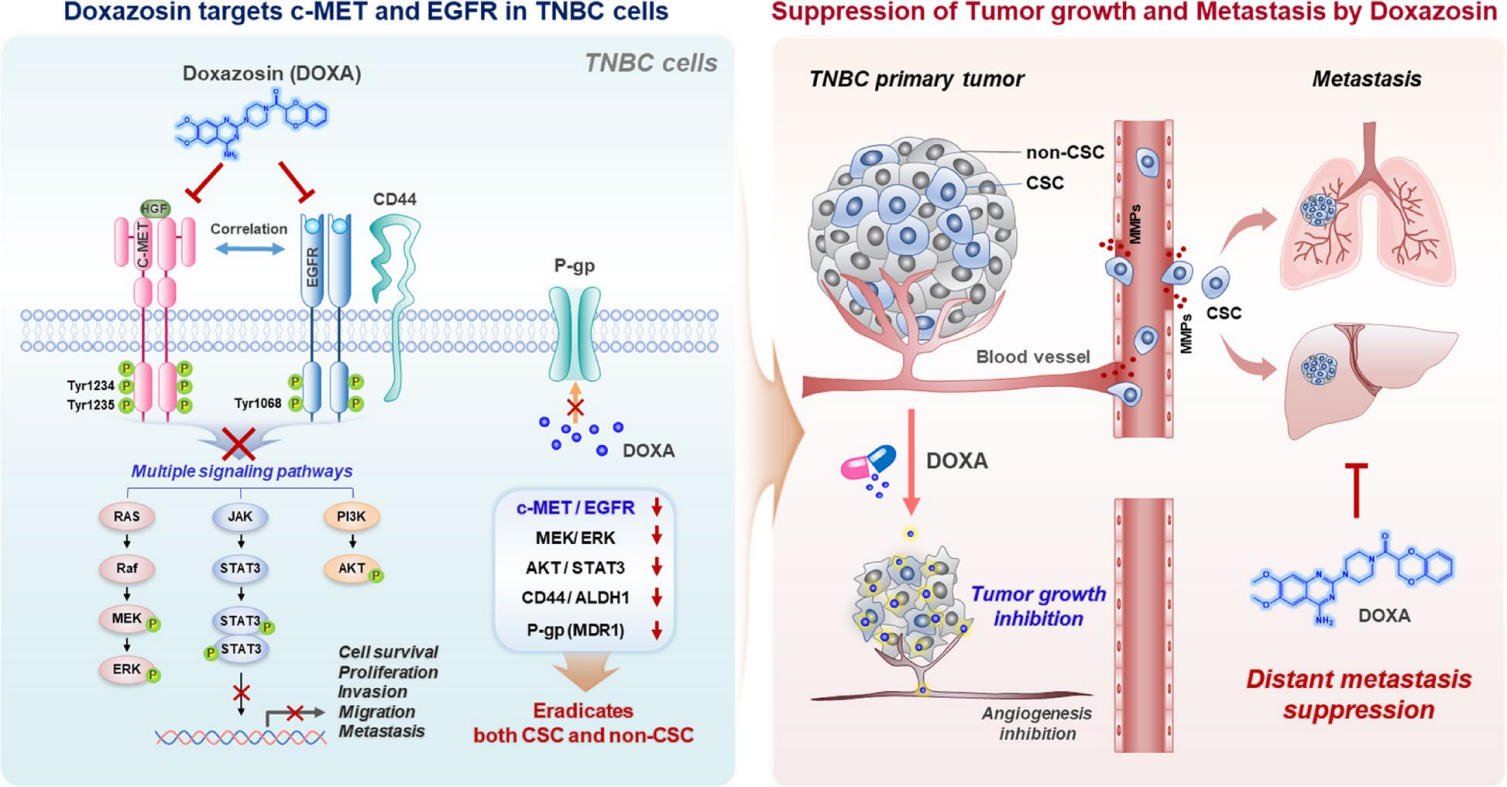
Hypothetical model illustrating the mechanism underlying DOXA’s ability to suppress tumor growth and metastasis in TNBC. DOXA effectively targets the c-MET and EGFR signaling pathways by directly binding to their tyrosine kinase domains. This interaction leads to apoptotic cell death and simultaneous suppression of multiple pro-survival pathways in TNBC cells. Furthermore, DOXA disrupts the interaction between EGFR and CD44, reducing CSC-like characteristics and downregulating stemness markers. In a CSC-enriched allograft mouse model, DOXA administration inhibits tumor growth, angiogenesis, and distant metastasis, accompanied by reduced levels of circulating VEGF, MMP-2, and MMP-9

Drug repurposing represents a viable strategy for improving overall survival for TNBC patients, particularly for those in lower socioeconomic classes, as clinical development risks relating to manufacturing, pharmacokinetics and safety parameters are largely absent. Our findings support the clinical application of DOXA as a safe and accessible treatment option for TNBC, particularly as both c-MET and EGFR are validated targets for neoplasms. DOXA’s dual targeting of c-MET and EGFR effectively inhibits multiple pro-survival pathways and disrupts the EGFR/CD44 axis, effectively eliminating both proliferating non-CSCs and dormant CSCs (Fig. 8). Further investigations into the clinical application of doxazosin in TNBC treatment are warranted.

### Supplementary Information

**Additional file 1: Supplementary Fig. S1. **Effect of DOXA on apoptosis in mouse TNBC 4T1 cells. (A-B) Cells were treated with DOXA (0–20 µM) for 48 h. (A) The sub-G1 population was quantified using flow cytometry (**p<0.01). (B) The proportion of early and late apoptotic cells in the presence or absence of DOXA was determined by annexin V/PI staining (***p<0.001). Results are presented as mean values ± SEM of at least three independent experiments and were analyzed by one-way ANOVA followed by Bonferroni’s multiple comparison test. **Supplementary Fig. S2. **Effect of DOXA on the expression of apoptosis-related proteins in 4T1 cells. Immunoblot analyses of PARP, cleaved caspase-3 and cleaved caspase-7 expression in 4T1 cells following exposure to DOXA (0-20 µM, 48 h). Quantitative graphs of protein content relative to GAPDH expression are shown in the right panel (**p<0.01). Results are presented as mean values ± SEM of at least three independent experiments and were analyzed by one-way ANOVA followed by Bonferroni’s multiple comparison test. **Supplementary Fig. S3. **Effect of amivantamab on cell viability in TNBC cells. (A) MDA-MB-231 and (B) BT549 cells were treated with various concentrations of amivantamab (1-1000 μg/ml) or control vehicle (DMSO) for 72 h. (C) Cell viability, 50% inhibitory concentration (IC50) and 95% confidence interval (CI95) values were determined by MTS assay (*p<0.0001). **Supplementary Fig. S4. **Immunoblotting analysis for c-MET expression in TNBC cells after treatment with DOXA, corresponding to Fig. 2D in the main text. Quantitative graphs represent the ratio of p-c-MET/total-MET expression levels in the presence or absence of DOXA (30-40 μM, 48 h) in MDA-MB-231 and BT549 cells (***p<0.001). Results are presented as mean values ± SEM of at least three independent experiments and were analyzed by one-way ANOVA followed by Bonferroni’s multiple comparison test. **Supplementary Fig. S5. **Effect of DOXA on p-glycoprotein mRNA expression in MDA-MB-231 cells. The relative mRNA expression of P-glycoprotein (P-gp) was analyzed by quantitative RT-PCR in MDA-MB-231 cells after treatment with DOXA (40 μM, 0-48 h). The quantitative graph represents the ratio of P-gp/GAPDH mRNA expression. Results are presented as mean values ± SEM of at least three independent experiments and were analyzed by one-way ANOVA followed by Bonferroni’s multiple comparison test. **Supplementary Fig. S6. **Immunoblot analysis of immunoprecipitated with EGFR for CD44 expression in TNBC cells after treatment with DOXA. MDA-MB-231 and BT549 cells were treated with DOXA (40 μM) for 24 h. Cell lysates were immunoprecipitated (IP) with anti-EGFR antibody and analyzed by immunoblotting (IB) with CD44 antibody. Whole lysate used for immunoprecipitation indicates input group and IgG means normal mouse immunoglobulin G. **Supplementary Fig. S7. **Impact of DOXA on BCSC-like property in 4T1 cells. (A-B) Cells were treated with DOXA (0-20 μM) for 48 h. (A) CD44^high^/CD24^low^ populations were determined by flow cytometry. The quantitative graph represents the percentage of CD44^high^/CD24^low^ populations (****p<0.0001). (B) Aldefluor-positivity was assessed and quantified (*p<0.05). DEAB was defined as the baseline of Aldefluor fluorescence with flow cytometry. Results are presented as mean ± SEM of at least three independent experiments and analyzed by one-way ANOVA followed by Bonferroni's multiple comparison test. **Supplementary Fig. S8. **Effect of DOXA on cell migration in 4T1 cells. (A-C) Cells treated with DOXA (0-40 μM) for 24 h. (A) The kinetic analysis of cell migration was determined using the IncuCyte™ Live-Cell Imaging System and quantified for the indicated time duration (*p<0.05). (B) The quantitative graph represents the relative wound density (%) in 4T1 cells at 24 h (*p<0.05). (C) Representative images of wound closure by cell migration at 0 and 24 h after treatment with DOXA (40 μM). The yellow dotted line indicates the edge of the scratched wound. Data were analyzed by one-way ANOVA followed by Bonferroni’s multiple comparison test. **Supplementary Fig. S9. **Influence of DOXA on CD49f expression in allograft tumors derived from 4T1 mammospheres. Tumor tissues were immunostained for CD49f (green) with DAPI (nuclei, blue). Quantitative graphs of signal intensities are shown in the right panel (****p<0.0001). The results are presented as mean ± SEM and data were analyzed by unpaired Student’s *t*-test.

**Additional file 2: Supplementary Table S1.** Sequences of primers used in RT-qPCR analysis. **Supplementary Table S2.** IC_50_ values of c-MET and EGFR inhibitors in TNBC cell lines. **Supplementary Table S3.** Prediction of protein-ligand binding affinity. **Supplementary Table S4.** Prediction of c-MET(6SD9)-ligand binding affinity. **Supplementary Table S5.** Prediction of EGFR(5CAP)-ligand binding affinity.

## Data Availability

All study data are included in the article and/or supplementary information.

## Abbreviations

ALDH1: Aldehyde dehydrogenase 1
AST: Aspartate aminotransferase
BUN: Blood urea nitrogen
CSC: Cancer stem cell
CD44: Cluster of differentiation 44
ERK: Extracellular signal-regulated kinase
EMT: Epithelial-mesenchymal transition
ECM: Extracellular matrix
GENT2: Gene Expression database of Normal and Tumor tissues 2
JAK: Janus tyrosine kinase
MAPK: Mitogen-activated protein kinase
MEK: Mitogen-activated protein kinase
MET: Mesenchymal-epithelial transition
MMP: Matrix metalloproteinase
METABRIC: The Molecular Taxonomy of Breast Cancer International Consortium
NANOG: Nanog homeobox
OCT4: Octamer-binding transcription factor 4
RTK: Receptor tyrosine kinase
SOX2: SRY-Box transcription factor 2
SMAD: Suppressor of mothers against decapentaplegic
TKIs: Tyrosine kinase inhibitors
TCGA: The Cancer Genome Atlas
VEGF: Vascular endothelial growth factor
