# Supplementary material for: Suppression of TNBC metastasis by doxazosin, a novel dual inhibitor of c-MET/EGFR
Source: J Exp Clin Cancer Res. 2023 Nov 4;42:292. doi: 10.1186/s13046-023-02866-z (PMC10625208; doi:10.1186/s13046-023-02866-z)
Supplement: Supplementary file 1 — Additional file 1: Supplementary Fig. S1. Effect of DOXA on apoptosis in mouse TNBC 4T1 cells. (A-B) Cells were treated with DOXA (0–20 µM) for 48 h. (A) The sub-G1 population was quantified using flow cytometry (**p<0.01). (B) The proportion of early and late apoptotic cells in the presence or absence of DOXA was determined by annexin V/PI staining (***p<0.001). Results are presented as mean values ± SEM of at least three independent experiments and were analyzed by one-way ANOVA followed by Bonferroni’s multiple comparison test. Supplementary Fig. S2. Effect of DOXA on the expression of apoptosis-related proteins in 4T1 cells. Immunoblot analyses of PARP, cleaved caspase-3 and cleaved caspase-7 expression in 4T1 cells following exposure to DOXA (0-20 µM, 48 h). Quantitative graphs of protein content relative to GAPDH expression are shown in the right panel (**p<0.01). Results are presented as mean values ± SEM of at least three independent experiments and were analyzed by one-way ANOVA followed by Bonferroni’s multiple comparison test. Supplementary Fig. S3. Effect of amivantamab on cell viability in TNBC cells. (A) MDA-MB-231 and (B) BT549 cells were treated with various concentrations of amivantamab (1-1000 μg/ml) or control vehicle (DMSO) for 72 h. (C) Cell viability, 50% inhibitory concentration (IC50) and 95% confidence interval (CI95) values were determined by MTS assay (*p<0.0001). Supplementary Fig. S4. Immunoblotting analysis for c-MET expression in TNBC cells after treatment with DOXA, corresponding to Fig. 2D in the main text. Quantitative graphs represent the ratio of p-c-MET/total-MET expression levels in the presence or absence of DOXA (30-40 μM, 48 h) in MDA-MB-231 and BT549 cells (***p<0.001). Results are presented as mean values ± SEM of at least three independent experiments and were analyzed by one-way ANOVA followed by Bonferroni’s multiple comparison test. Supplementary Fig. S5. Effect of DOXA on p-glycoprotein mRNA expression in MDA-MB-231 ce [file 13046_2023_2866_MOESM1_ESM.docx]

**Suppression of TNBC metastasis by doxazosin, a novel dual inhibitor of c-MET/EGFR**

Seongjae Kim^1,2^, Jung Min Park^1,2^, Soeun Park^1,2^, Eunsun Jung^1,2^, Dongmi Ko^1,2^, Minsu Park^1,2^, Juyeon Seo^1,2^, Kee Dal Nam^1,3^, Yong Koo Kang^1,3^, Kyoungmin Lee^1,3^, Lee Farrand^4^, Yoon-Jae Kim^1,2,3,*^, Ji Young Kim^1,3,**^ and Jae Hong Seo^1,2,3,***^

**Supplementary information**

**Supplementary Figures and Legends (Fig. S1-S9)**

***Supplementary Fig. S1***

**
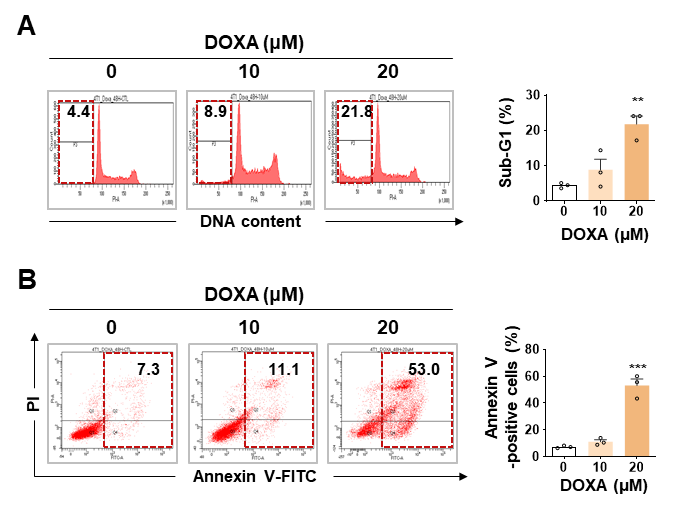
**

**Supplementary Fig. S1. Effect of DOXA on apoptosis in mouse TNBC 4T1 cells.** (A-B) Cells were treated with DOXA (0–20 µM) for 48 h. (A) The sub-G1 population was quantified using flow cytometry (***p*<0.01). (B) The proportion of early and late apoptotic cells in the presence or absence of DOXA was determined by annexin V/PI staining (****p*<0.001). Results are presented as mean values ± SEM of at least three independent experiments and were analyzed by one-way ANOVA followed by Bonferroni’s multiple comparison test.

***Supplementary Fig. S2***


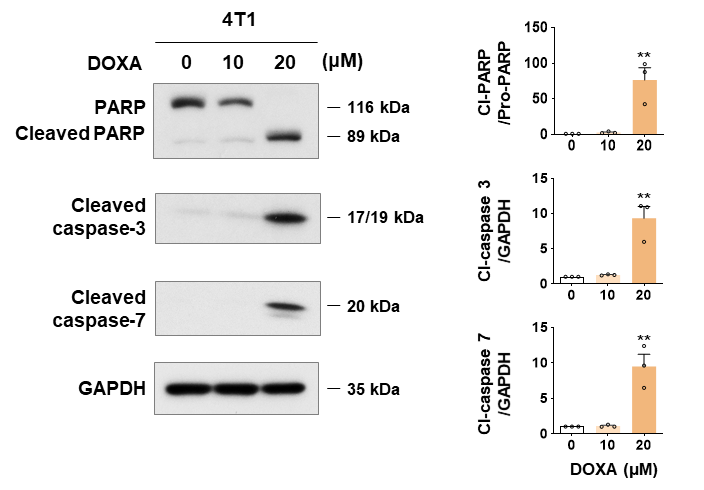


**Supplementary Fig. S2. Effect of DOXA on the expression of apoptosis-related proteins in 4T1 cells.** Immunoblot analyses of PARP, cleaved caspase-3 and cleaved caspase-7 expression in 4T1 cells following exposure to DOXA (0-20 µM, 48 h). Quantitative graphs of protein content relative to GAPDH expression are shown in the right panel (***p*<0.01). Results are presented as mean values ± SEM of at least three independent experiments and were analyzed by one-way ANOVA followed by Bonferroni’s multiple comparison test.

***Supplementary Fig. S3***

***
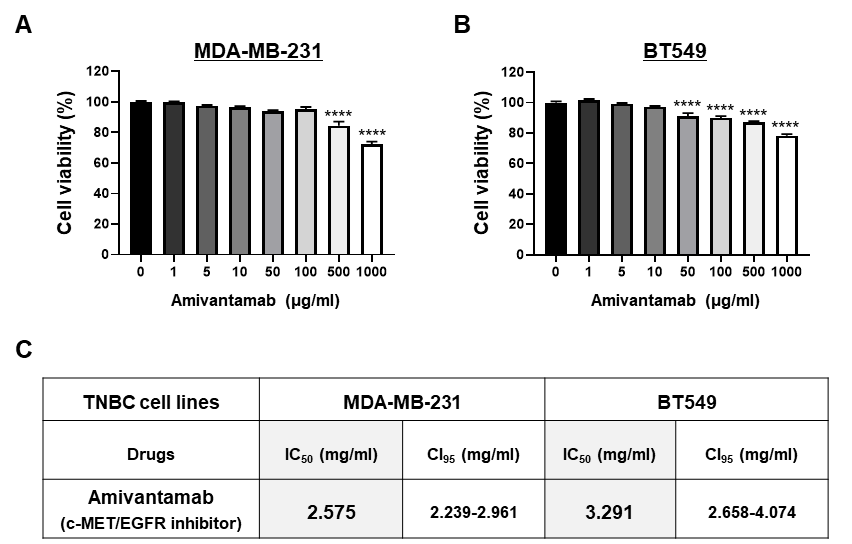
***

**Supplementary Fig. S3. Effect of amivantamab on cell viability in TNBC cells.** (A) MDA-MB-231 and (B) BT549 cells were treated with various concentrations of amivantamab (1-1000 μg/ml) or control vehicle (DMSO) for 72 h. (C) Cell viability, 50% inhibitory concentration (IC50) and 95% confidence interval (CI95) values were determined by MTS assay (*p<0.0001).

***Supplementary Fig. S4***


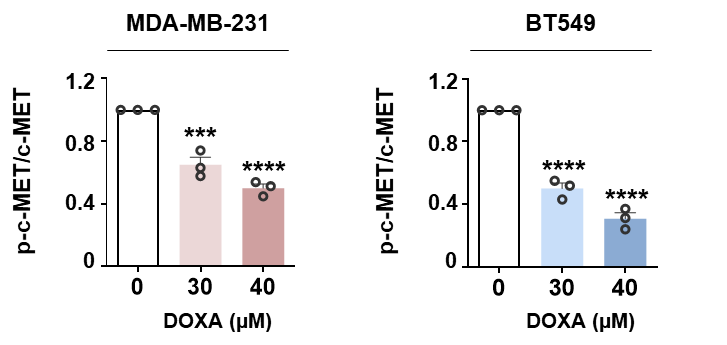


**Supplementary Fig. S4. Immunoblotting analysis for c-MET expression in TNBC cells after treatment with DOXA,** **corresponding to Fig. 2D in the main text.** Quantitative graphs represent the ratio of p-c-MET/total-MET expression levels in the presence or absence of DOXA (30-40 μM, 48 h) in MDA-MB-231 and BT549 cells (****p*<0.001). Results are presented as mean values ± SEM of at least three independent experiments and were analyzed by one-way ANOVA followed by Bonferroni’s multiple comparison test.

***Supplementary Fig. S5***


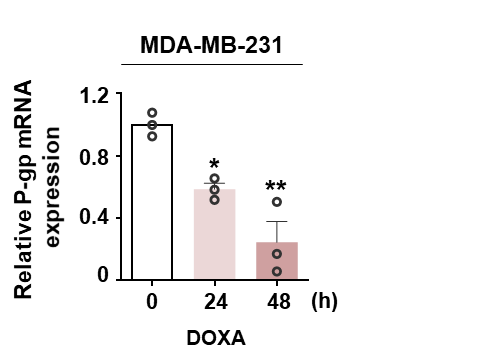


**Supplementary Fig. S5. Effect of DOXA on p-glycoprotein mRNA expression in MDA-MB-231 cells.** The relative mRNA expression of P-glycoprotein (P-gp) was analyzed by quantitative RT-PCR in MDA-MB-231 cells after treatment with DOXA (40 μM, 0-48 h). The quantitative graph represents the ratio of P-gp/GAPDH mRNA expression. Results are presented as mean values ± SEM of at least three independent experiments and were analyzed by one-way ANOVA followed by Bonferroni’s multiple comparison test.

***Supplementary Fig. S6***


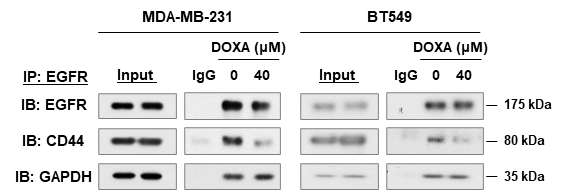


**Supplementary Fig. S6. Immunoblot analysis of immunoprecipitated with EGFR for CD44 expression in TNBC cells after treatment with DOXA.** MDA-MB-231 and BT549 cells were treated with DOXA (40 μM) for 24 h. Cell lysates were immunoprecipitated (IP) with anti-EGFR antibody and analyzed by immunoblotting (IB) with CD44 antibody. Whole lysate used for immunoprecipitation indicates input group and IgG means normal mouse immunoglobulin G.

***Supplementary Fig. S7***


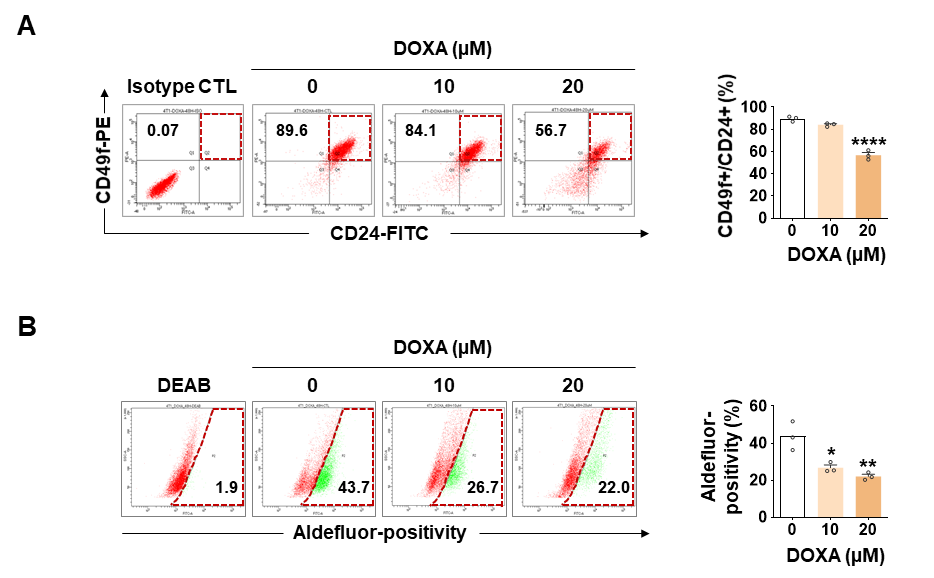


**Supplementary Fig. S7. Impact of DOXA on BCSC-like property in 4T1 cells.** (A-B) Cells were treated with DOXA (0-20 μM) for 48 h. (A) CD44^high^/CD24^low^ populations were determined by flow cytometry. The quantitative graph represents the percentage of CD44^high^/CD24^low^ populations (*****p*<0.0001). (B) Aldefluor-positivity was assessed and quantified (**p*<0.05). DEAB was defined as the baseline of Aldefluor fluorescence with flow cytometry. Results are presented as mean ± SEM of at least three independent experiments and analyzed by one-way ANOVA followed by Bonferroni's multiple comparison test.

***Supplementary Fig. S8***


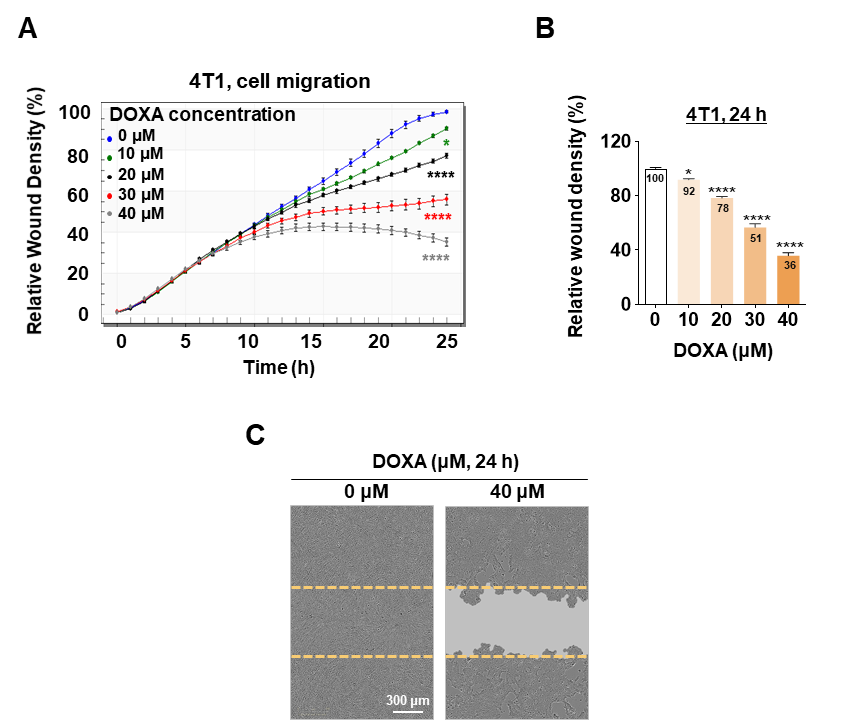


**Supplementary Fig. S8. Effect of DOXA on cell migration in 4T1 cells.** (A-C) Cells treated with DOXA (0-40 μM) for 24 h. (A) The kinetic analysis of cell migration was determined using the IncuCyte™ Live-Cell Imaging System and quantified for the indicated time duration (**p*<0.05). (B) The quantitative graph represents the relative wound density (%) in 4T1 cells at 24 h (**p*<0.05). (C) Representative images of wound closure by cell migration at 0 and 24 h after treatment with DOXA (40 μM). The yellow dotted line indicates the edge of the scratched wound. Data were analyzed by one-way ANOVA followed by Bonferroni’s multiple comparison test.

***Supplementary Fig. S9***


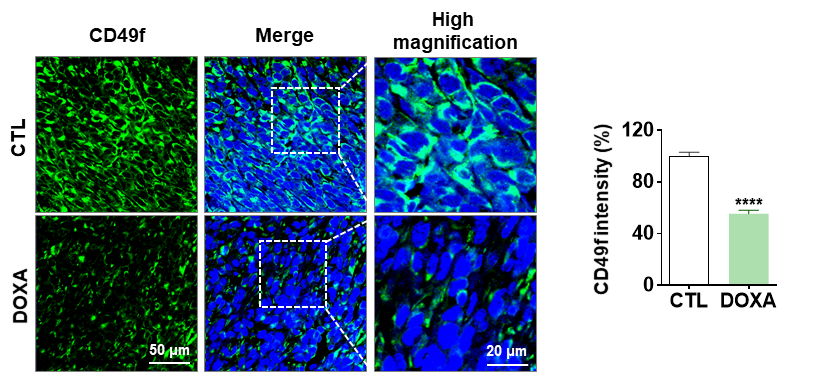


**Supplementary Fig. S9. Influence of DOXA on CD49f expression in allograft tumors derived from 4T1 mammospheres.** Tumor tissues were immunostained for CD49f (green) with DAPI (nuclei, blue). Quantitative graphs of signal intensities are shown in the right panel (*****p*<0.0001). The results are presented as mean ± SEM and data were analyzed by unpaired Student’s *t*-test.
