# Supplementary material for: Suppression of TNBC metastasis by doxazosin, a novel dual inhibitor of c-MET/EGFR
Source: J Exp Clin Cancer Res. 2023 Nov 4;42:292. doi: 10.1186/s13046-023-02866-z (PMC10625208; doi:10.1186/s13046-023-02866-z)
Supplement: Supplementary file 2 — Additional file 2: Supplementary Table S1. Sequences of primers used in RT-qPCR analysis. Supplementary Table S2. IC50 values of c-MET and EGFR inhibitors in TNBC cell lines. Supplementary Table S3. Prediction of protein-ligand binding affinity. Supplementary Table S4. Prediction of c-MET(6SD9)-ligand binding affinity. Supplementary Table S5. Prediction of EGFR(5CAP)-ligand binding affinity. [file 13046_2023_2866_MOESM2_ESM.docx]

**Suppression of TNBC metastasis by doxazosin, a novel dual inhibitor of c-MET/EGFR**

Seongjae Kim^1,2^, Jung Min Park^1,2^, Soeun Park^1,2^, Eunsun Jung^1,2^, Dongmi Ko^1,2^, Minsu Park^1,2^, Juyeon Seo^1,2^, Kee Dal Nam^1,3^, Yong Koo Kang^1,3^, Kyoungmin Lee^1,3^, Lee Farrand^4^, Yoon-Jae Kim^1,2,3,*^, Ji Young Kim^1,3,**^ and Jae Hong Seo^1,2,3,***^

**Supplementary information**

**Supplementary Table**

Supplementary Table S1. Sequences of primers used in RT-qPCR analysis

| **Gene** | **Gene ID** | **Primer** | **Sequence (5’ to 3’)** | **NCBI sequence** |
| --- | --- | --- | --- | --- |
| Cyclin D1 | CCND1 | F | CAATGACCCCGCACGATTTC | NM_053056 |
|  |  | R | CATGGAGGGCGGATTGGAA |  |
| Survivin | BIRC5 | F | AGGACCACCGCATCTCTACAT | NM_001168 |
|  |  | R | AAGTCTGGCTCGTTCTCAGTG |  |
| Vimentin | VIM | F | GACGCCATCAACACCGAGTT | NM_003380 |
|  |  | R | CTTTGTCGTTGGTTAGCTGGT |  |
| VEGF-A | VEGFA | F | AGGGCAGAATCATCACGAAGT | NM_001025366 |
|  |  | R | AGGGTCTCGATTGGATGGCA |  |
| MMP-2 | MMP2 | F | TACAGGATCATTGGCTACACACC | NM_004530 |
|  |  | R | GGTCACATCGCTCCAGACT |  |
| MMP-9 | MMP9 | F | AGACCTGGGCAGATTCCAAAC | NM_004994 |
|  |  | R | CGGCAAGTCTTCCGAGTAGT |  |
| Smad-3 | SMAD3 | F | CCATCTCCTACTACGAGCTGAA | NM_005902 |
|  |  | R | CACTGCTGCATTCCTGTTGAC |  |
| Smad-4 | SMAD4 | F | CTCATGTGATCTATGCCCGTC | NM_005359 |
|  |  | R | AGGTGATACAACTCGTTCGTAGT |  |
| P-gp | ABCB1 | F | GGGAGCTTAACACCCGACTTA | NM_001348945 |
|  |  | R | GCCAAAATCACAAGGGTTAGCTT |  |

Supplementary Table S2. IC_50_ values of c-MET and EGFR inhibitors in TNBC cell lines

|  | TNBC  cell lines | MDA-MB-231 | | BT549 | | 4T1 | |
| --- | --- | --- | --- | --- | --- | --- | --- |
|  | **Drugs** | **IC_50_ (µM)** | **CI_95_ (µM)** | **IC_50_ (µM)** | **CI_95_ (µM)** | **IC_50_ (µM)** | **CI_95_ (µM)** |
| c-MET inhibitor | **Crizotinib** | **3.309** | **2.730-4.011** | **8.119** | **5.611-11.75** | **3.173** | **2.340-4.304** |
|  | **Capmatinib** | **124.6** | **89.13-174.1** | **23.88** | **13.06-43.66** | **49.86** | **37.42-66.42** |
|  | **Tepotinib** | **5.974** | **4.733-7.540** | **10.13** | **6.686-15.34** | **1.530** | **1.179-1.985** |
| EGFR inhibitor | **Osimertinib** | **4.055** | **2.904-5.661** | **5.749** | **3.636-9.090** | **4.315** | **2.950-6.310** |
|  | **Lazertinib** | **10.93** | **7.983-14.96** | **7.408** | **4.989-11.00** | **11.16** | **8.595-14.48** |
| c-MET/EGFR inhibitor | **Doxazosin** | **26.52** | **20.40-34.49** | **27.32** | **21.13-35.33** | **7.530** | **5.982-9.478** |

**Supplementary Table S2. Effect of c-MET and EGFR inhibitors on cell viability in TNBC cells.** MDA-MB-231, BT549 and 4T1 cells were treated with various concentrations of c-MET inhibitors (crizotinib, capmatinib and tepotinib), EGFR inhibitors (osimertinib and lazertinib) or control vehicle (DMSO) for 48 h. Cell viability, 50% inhibitory concentration (IC_50_) and 95% confidence interval (CI_95_) values were determined by MTS assay.

Supplementary Table S3. Prediction of protein-ligand binding affinity

| Protein  (PDB) | Ligand  (PubChem) | Affinity Score  (kcal/mol) | Total Energy  (kcal/mol) | Interaction Energy  (kcal/mol) |
| --- | --- | --- | --- | --- |
| EGFR  (5CAP) | Doxazosin  (3157) | -9.004 | 52.933 | -37.328 |
| c-MET  (6SD9) | Doxazosin  (3157) | -9.226 | 55.335 | -37.413 |

DockThor server: <https://dockthor.lncc.br/v2/>

Supplementary Table S4. Prediction of c-MET(6SD9)-ligand binding affinity

| Protein  (PDB) | Ligand  (PubChem) | Affinity Score  (kcal/mol) | Total Energy  (kcal/mol) | Interaction Energy  (kcal/mol) |
| --- | --- | --- | --- | --- |
| c-MET  (6SD9) | Tepotinib  (25171648) | -9.793 | 82.944 | -43.772 |
|  | Capmatinib  (25145656) | -9.744 | 60.586 | -43.142 |
|  | Crizotinib  (11626560) | -9.179 | 57.494 | -38.385 |
|  | Doxazosin  (3157) | -9.226 | 55.335 | -37.413 |

DockThor server: <https://dockthor.lncc.br/v2/>

Supplementary Table S5. Prediction of EGFR(5CAP)-ligand binding affinity

| Protein  (PDB) | Ligand  (PubChem) | Affinity Score  (kcal/mol) | Total Energy  (kcal/mol) | Interaction Energy  (kcal/mol) |
| --- | --- | --- | --- | --- |
| EGFR  (5CAP) | Osimertinib  (71496458) | -8.947 | 86.884 | -37.868 |
|  | Lazertinib  (121269225) | -9.743 | 128.226 | -43.989 |
|  | Doxazosin  (3157) | -9.004 | 52.933 | -37.328 |

DockThor server: <https://dockthor.lncc.br/v2/>
